# Inhibition of Citrinin-Induced Apoptotic Biochemical Signaling in Human Hepatoma G2 Cells by Resveratrol

**DOI:** 10.3390/ijms10083338

**Published:** 2009-07-29

**Authors:** Chia-Chi Chen, Wen-Hsiung Chan

**Affiliations:** Department of Bioscience Technology and Center for Nanotechnology, Chung Yuan Christian University, Chung Li, 32023, Taiwan; E-Mail: cha_chi_chen@yahoo.com.tw (C.-C.C.)

**Keywords:** resveratrol, citrinin, ROS, JNK, apoptosis

## Abstract

The mycotoxin citrinin (CTN), a natural contaminant in foodstuffs and animal feeds, exerts cytotoxic and genotoxic effects on various mammalian cells. CTN causes cell injury, including apoptosis, but its precise regulatory mechanisms of action are currently unclear. Resveratrol, a member of the phytoalexin family found in grapes and other dietary plants, possesses antioxidant and anti-tumor properties. In the present study, we examined the effects of resveratrol on apoptotic biochemical events in Hep G2 cells induced by CTN. Resveratrol inhibited CTN-induced ROS generation, activation of JNK, loss of mitochondrial membrane potential (MMP), as well as activation of caspase-9, caspase-3 and PAK2. Moreover, resveratrol and the ROS scavengers, NAC and α-tocopherol, abolished CTN-stimulated intracellular oxidative stress and apoptosis. Active JNK was required for CTN-induced mitochondria-dependent apoptotic biochemical changes, including loss of MMP, and activation of caspases and PAK2. Activation of PAK2 was essential for apoptosis triggered by CTN. These results collectively demonstrate that CTN stimulates ROS generation and JNK activation for mitochondria-dependent apoptotic signaling in Hep G2 cells, and these apoptotic biochemical events are blocked by pretreatment with resveratrol, which exerts antioxidant effects.

## 1. Introduction

Several fungal species belonging to the genera *Penicillium* and *Monascus* produce secondary metabolites that include the mycotoxin citrinin (CTN). CTN has been identified as a contaminant in various types of food, including corn, wheat, rice, barley and nuts, and is associated with environmental and human health injuries [1,2]. Experiments with cell lines and animals models show that CTN triggers nephropathy and hepatotoxicity [3,4], as well as renal adenoma formation [5]. However, the molecular mechanisms underlying CTN-mediated biological functions and cytotoxicities have yet to be established.

Recent studies suggest that the cytotoxicity of CTN may be due to its ability to trigger apoptosis [6–8]. Numerous chemical and physical treatments capable of inducing apoptosis stimulate oxidative stress via intracellular ROS generation [9–11], suggesting a close relationship between oxidative stress and apoptosis. While the precise molecular mechanisms of apoptosis have not been clearly defined as yet, a number of cysteine proteases, designated ‘caspases’ [12,13] and Bcl-2 family proteins [14] appear to play important roles in this process. These proteins control mitochondrial membrane potential changes and release of mitochondrial cytochrome C by modulating outer membrane permeability. In addition, changes in protein kinase activity are observed during apoptosis in a variety of cell types [15], indicating that protein phosphorylation is involved in cell death regulation. In particular, the c-Jun N-terminal kinase (JNK) acts as a key component in regulating entry into apoptosis in several cell types [16–20]. Another enzyme, PAK (p21-activated kinase), which is additionally stimulated during apoptosis is implicated in signaling events [20–26]. While the direct downstream substrates of PAKs are largely unknown, earlier studies indicate that these enzymes act as upstream regulators of the JNK and p38 MAPK pathways [25,27–29]. We previously showed that PAK2 is required for photodynamic treatment (PDT)- and methylglyoxal-induced apoptosis [25,30]. While PAK2 clearly plays an important role in apoptotic signaling, further investigations are required to elucidate its direct downstream substrates and precise regulatory mechanisms.

Resveratrol, a member of the phytoalexin family found in grapes and other dietary plants, inhibits tumor initiation and progression [31–33]. Resveratrol exerts a wide range of pharmacological effects, including prevention of heart disorders, blocking of lipoprotein oxidation, and inhibition of platelet aggregation [34–36]. The anti-tumor properties of resveratrol have been attributed to its antioxidant activity and ability to inhibit cyclooxygenase 1 and 2 [31,37], along with the capacity to induce cell cycle arrest and apoptosis [38–43].

To clarify whether resveratrol is effective in preventing CTN-induced cell injury, we examined its effects on apoptosis induced by the mycotoxin. Pretreatment with resveratrol inhibited CTN-induced mitochondria-dependent apoptotic biochemical alterations in Hep G2 cells, including mitochondrial membrane potential (MMP) changes, caspase-9 and -3 activation, PAK2 activation, and DNA fragmentation. The inhibitory effect of resveratrol on CTN-induced apoptosis was additionally linked to its ability to attenuate ROS formation.

## 2. Results and Discussion

**Figure 1.**
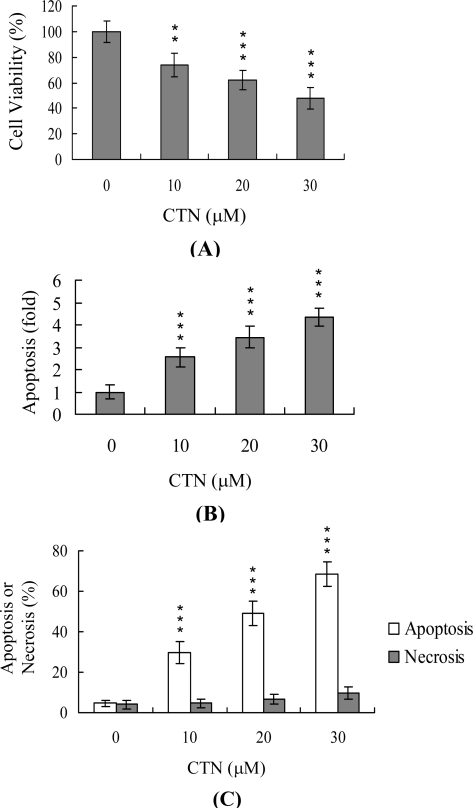

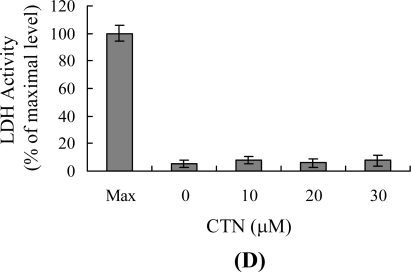
Effect of CTN on viability of Hep G2 cells. Hep G2 cells were treated with the indicated doses of citrinin (CTN) for 24 h or left untreated. Cell viability was determined with the MTT assay (A) and apoptosis detected with the ELISA kit (B). (C) The percentage of apoptotic and necrotic cells was determined by staining with propidium iodide and Hoechst 33342. (D) Activity of LDH released in the culture medium of Hep G2 cells after treatment with various concentration of CTN. Data are expressed as a percentage of the maximal level (Max) of LDH activity determined after total cell lysis. Values are presented as means ± SD of six determinations. **P<0.01 and ***P<0.001 versus value of the control (without CTN treatment) group.

Hep G2 cells were treated with various doses of CTN at 37 ^o^C for 24 h, and cell viability and apoptosis examined. As shown in Figure 1A, CTN reduced cell viability in a dose-dependent manner. The next step was to determine whether CTN-induced cell death was due to apoptosis. ELISA findings revealed that CTN induced a significant increase in DNA fragmentation in a concentration-dependent manner (Figure 1B). The percentages of apoptotic and necrotic cells were further analyzed by propidium iodide and Hoechst 33342 staining, and necrotic events assessed by evaluating LDH activity in the culture medium. As shown in Figure 1C, the percentage of apoptotic cells increased significantly at CTN concentrations higher than 10 μM. However, under these conditions, the necrotic cell population remained relatively low (Figures 1C and 1D).

**Figure 2.**
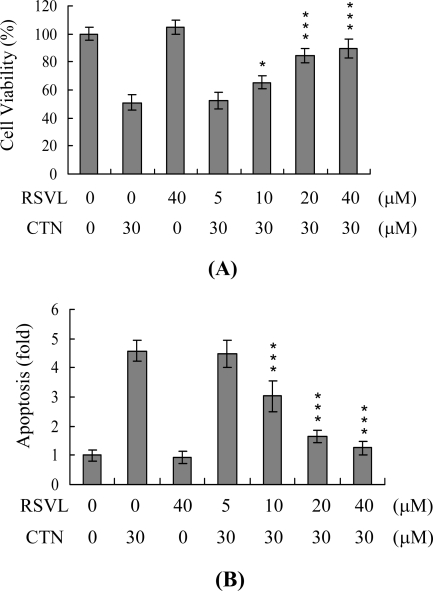
Resveratrol prevents CTN-induced cell death. Hep G2 cells were incubated with the indicated concentrations of resveratrol (RSVL) for 1 h or left untreated. Next, cells were treated with or without CTN (30 μM) for another 24 h. Cell viability (A) and apoptosis were measured (B). The number of viable cells in the control samples was set as 100%. *P<0.05 and **P<0.001 versus value of the group treated with 30 μM CTN.

Next, the effect of resveratrol on CTN-induced apoptosis was examined. Hep G2 cells were incubated with various doses of resveratrol or treated with 30 μM CTN after preincubation with resveratrol, and cell viability determined. Resveratrol alone (40 μM) had no effect on cell viability, but inhibited CTN-induced cell apoptosis in a dose-dependent manner (Figures 2A and 2B). The results clearly demonstrate that resveratrol is a potent inhibitor of CTN-induced apoptosis.

**Figure 3.**
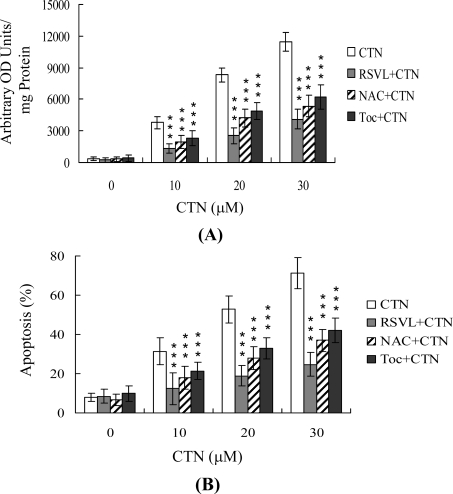
Resveratrol attenuates CTN-induced oxidative stress. Hep G2 cells were preloaded with 20 μM DCF-DA for 1 h. Cells were left untreated or treated with 20 μM resveratrol (RSVL), 400 μM N-acetyl cysteine (NAC), or 300 μM α-tocopherol (Toc) as indicated for 1 h, followed by incubation of 10–30 μM CTN for another 24 h. (A) ROS generation was assayed by DCF-DA, and expressed as absorbance/mg of protein. (B) Apoptosis was detected with the ELISA kit. Values are presented as means ± SD of eight determinations. ***P<0.001 versus value of the group treated with CTN.

Earlier studies report that CTN promotes oxidative stress in cells [7,20], and ROS are effective apoptotic inducers. Accordingly, we examined ROS formation in CTN-treated Hep G2 cells and the effects of resveratrol on this process, using DCF-DA as the detection reagent. As shown in Figure 3A, CTN augmented the intracellular ROS content in Hep G2 cells, which was significantly attenuated upon pre-treatment with resveratrol. Two frequently used ROS scavengers, N-acetyl cysteine (NAC) and α-tocopherol, were tested in parallel. Pretreatment with NAC (400 μM) and α-tocopherol (300 μM) additionally led to suppression of CTN-stimulated intracellular ROS levels and apoptosis (Figures 3A and 3B). Interestingly, under our assay conditions, resveratrol appeared to be the most potent blocker of CTN-induced oxidative stress and apoptosis (Figures 3A and 3B). Based on these results, we propose that resveratrol effectively prevents CTN-induced apoptosis via its ability to act as a ROS scavenger.

**Figure 4.**
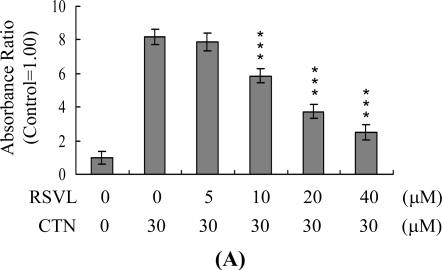

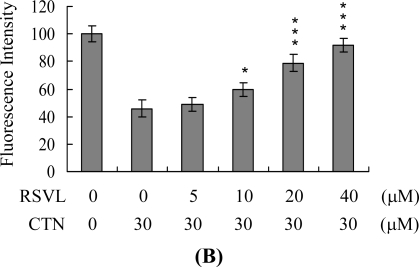
Resveratrol inhibits CTN-induced JNK activation and loss of mitochondrial membrane potential (MMP). Hep G2 cells were preincubated with the indicated concentrations of resveratrol (RSVL) for 1 h, followed by CTN (30 μM) for 24 h. (A) JNK/AP-1 activity was evaluated by ELISA detection of phosphorylated c-Jun. Results are expressed in relation to control values, which were arbitrarily set as 1.00. (B) MMP changes were analyzed using 40 nM DiOC_6_(3). Values are presented as means ± SD of five determinations. *P<0.05 and ***P<0.001 versus the CTN-treated group.

JNK activation and loss of MMP are directly associated with apoptosis [11,17,18], and these events are observed in CTN-treated embryonic stem cells and osteoblasts [7,29]. Accordingly, we examined the effects of resveratrol on CTN-induced JNK activity in Hep G2 cells. As shown in Figure 4A, resveratrol inhibited CTN-induced JNK activity in a dose-dependent manner. Moreover, CTN induced a significant loss of MMP, compared with non-treated control cells (Figure 4B), which was suppressed by pretreatment with Resveratrol in a dose-dependent manner (Figure 4B).

**Figure 5.**
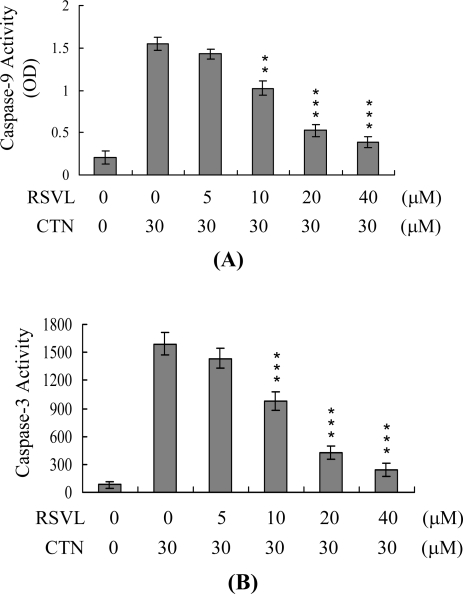

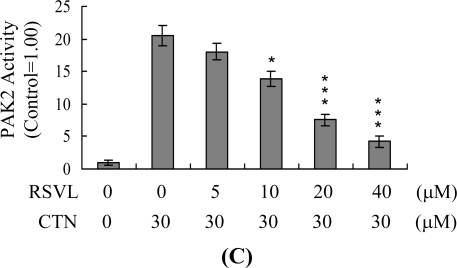
Resveratrol inhibits CTN-induced activation of caspases-3, -9, and PAK2. Hep G2 cells were preincubated with the indicated concentrations of resveratrol (RSVL) for 1 h, followed by CTN (30 μM) for 24 h. (A) Caspase-9 activity was assayed using the Colorimetric Caspase-9 Assay Kit (Calbiochem). (B) Cell extracts (60 μg) were analyzed for caspase-3 activity using Z-DEVD-AFC as the substrate. (C) The C-terminal catalytic fragment of PAK2 was immunoprecipitated, and kinase activities assayed using myelin basic protein as the substrate. Values are presented as means ± SD of six determinations. *P<0.05, **P<0.01 and ***P<0.001 versus the CTN-treated group.

To further explore the apoptotic signaling pathway of CTN-induced cell death, we employed *in vitro* ELISA to monitor activation of caspases-9 and -3, which are involved in apoptosis of multiple cell types triggered by a variety of stimuli [10,44–46]. CTN treatment of Hep G2 cells stimulated activities of both caspase-9 (Figure 5A) and caspase-3 (Figure 5B). However, caspase-8 activities and Fas protein expression levels were not significantly different between CTN-treated and untreated cells (data not shown). In view of previous studies reporting that p21-activated kinase 2 (PAK2) activated during apoptosis is involved in signaling events [20,23–25,30,47,48], possibly via caspase-3-directed proteolysis [23–25,30], we examined whether PAK2 is activated in CTN-treated Hep G2 cells. Immunoprecipitation kinase activity assays disclosed that CTN activates PAK2 (Figure 5C). These results collectively indicate that caspases-9 and -3 and PAK2 are involved in CTN-induced apoptosis of Hep G2 cells. Treatment of cells with resveratrol at concentrations higher than 10 μM led to significant inhibition of the apoptotic biochemical changes induced by CTN (Figures 5A–5C).

**Figure 6.**
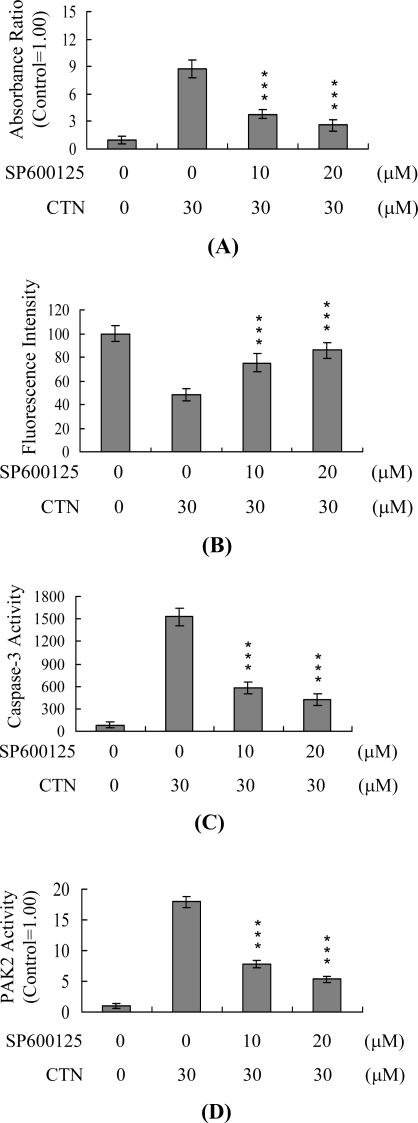

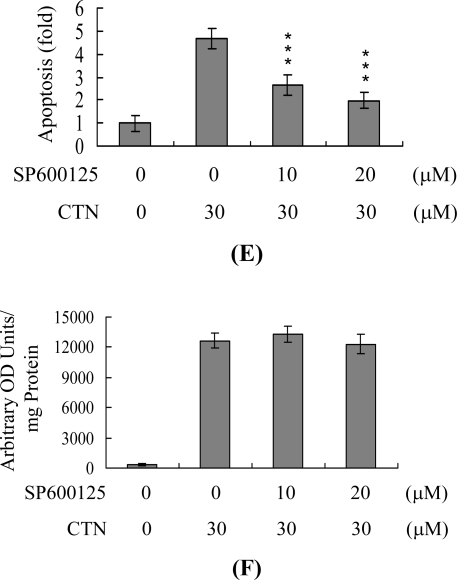
Effects of SP600125 on CTN-induced JNK activation, loss of MMP, activation of caspase-3 and PAK2, apoptosis, and ROS generation. Hep G2 cells were preincubated with various concentrations of SP600125 at 37 ^o^C for 1 h, and treated with 30 μM CTN for another 24 h. (A) JNK/AP-1 activity was evaluated by ELISA detection of phosphorylated c-Jun. MMP changes (B), activation of caspase-3 (C) and PAK2 (D), apoptosis (E), and ROS generation (F) were measured, as described in Figures 3–5. Values are presented as means ± SD of five determinations. ***P<0.001 versus the CTN-treated group.

To further determine whether activation of JNK, caspase-3 and PAK2 and ROS production are interlinked during CTN-induced apoptosis, we examined the effects of the specific JNK inhibitor, SP600125 [49], on CTN-treated Hep G2 cells. SP600125 pretreatment reduced CTN-stimulated JNK activity by ~40% (Figure 6A), in association with a significant decrease in loss of MMP (Figure 6B), activation of caspase-3 (Figure 6C) and PAK2 (Figure 6D), and apoptosis (Figure 6E). However, the ROS levels remained unaffected (Figure 6F). These findings indicate that ROS generation occurs upstream of JNK, which occurs prior to caspase-3 and PAK2 activation, and subsequent apoptotic biochemical changes during CTN-induced apoptosis.

**Figure 7.**
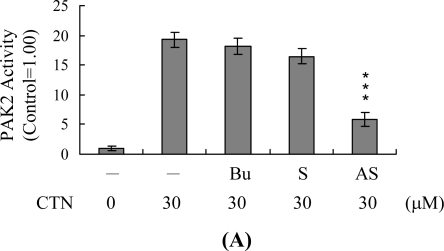

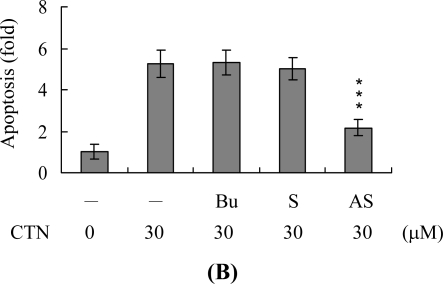
Effects of antisense oligonucleotides against PAK2 on CTN-induced apoptosis in Hep G2 cells. Hep G2 cells were incubated with buffer (Bu), 70 μM PAK2 sense (S) or antisense (AS) oligonucleotides in the presence of Lipofectamine for 72 h, followed by CTN (30 μM) for another 24 h. (A) PAK2 was immunoprecipitated and kinase activities assayed using myelin basic protein (MBP) as the substrate. (B) Apoptosis was measured with the Cell Death Detection ELISA kit (TUNEL assay kit). Values are presented as means ± SD of six determinations. ***P<0.001 versus cells treated with CTN alone.

To further determine the functional role of PAK2 in CTN-induced apoptosis, we incubated Hep G2 cells with antisense or sense oligonucleotides against PAK2 for 3 days, followed by CTN treatment. Cells were analyzed by immunoprecipitation. Pre-incubation of cells with an antisense oligonucleotide against PAK2 led to a significant decrease in its activations levels to approximately 35–40% considering CTN-treated cells (Figure 7A). This decrease in PAK2 activation was associated with a significant decline in CTN-induced apoptosis (Figure 7B), but no changes were detected in ROS production levels, JNK activation, loss of MMP, and activation of caspase-3 (data not shown). These results strongly indicate that CTN promotes caspase-3 and PAK2 activation through the mitochondria-dependent apoptotic signal pathway, and PAK2 plays an important role in CTN-induced apoptosis of Hep G2 cells.

Foodstuffs and animal feeds are often contaminated with various fungal toxins, including the mycotoxin CTN, which has been identified in cereal grains and fermented maize dough at levels of about 180~580 ng/g [50]. CTN contamination has additionally been reported in *Monascus* fermentation products [51,52] used as food colorants and flavor enhancers in the Orient and as dietary supplements believed to prevent heart disease and decrease plasma triglyceride and cholesterol levels [53]. A recent study estimated CTN levels of 0.28 to 6.29 μg/g in lipid extracts from commercialized *Monascus* products [51], while another report showed that treatment of human HEK 293 cells with lipid extracts of *Monascus* products (60 μM CTN) for 72 h induced over 50% cell death [51]. In view of these findings, we examined the effects of 10–30 μM CTN (~1.25–3.75 μg/g in culture medium) on Hep G2 cells *in vitro*. The treatment dosage of CTN in the present study reflects its concentration in contaminated foods.

Mechanistically, CTN directly evoked intracellular oxidative stress (Figure 3A), leading to ROS-mediated apoptosis in Hep G2 cells (Figure 3B). Since the addition of specific compounds, such as hydrogen peroxide, to commonly utilized cell culture media triggers ROS generation [54,55], we co-incubated CTN and culture medium, and measured ROS levels using the ferrous iron oxidation-xylenol orange method [54]. No artifactual ROS generation was evident under these conditions (data not shown). In addition, resveratrol and two well-known ROS scavengers, NAC and α-tocopherol, effectively prevented CTN-induced ROS generation and apoptosis in Hep G2 cells (Figure 3). Our results collectively demonstrate that CTN triggers apoptosis in Hep G2 cells via ROS generation, which stimulates downstream apoptotic processes.

The inhibitory effect of resveratrol on apoptotic biochemical changes triggered by several stimuli is attributed to its antioxidant properties [56–58]. Oxidative stress is a recognized stimulator of cell responses, such as apoptosis. Antioxidants protect cells against apoptosis induced by various stimuli that exert both direct and indirect oxidant effects. The antioxidant and anti-inflammatory properties of resveratrol may be due to cyclooxygenase inhibition [31]. A recent report shows that resveratrol exerts a powerful antioxidant effect on multiple ROS (O_2_^−^ and H_2_O_2_) production in macrophage cells subjected to lipopolysaccharide (LPS)- or phorbol ester (PMA) treatment. O_2_^−^ production by LPS- or PMA-treated phagocytic cells occurs via the NADPH oxidase pathway [59]. Here, we show that resveratrol attenuates CTN-induced intracellular ROS formation, supporting the hypothesis that the compound suppresses apoptosis by inhibiting NADPH oxidase activation, consequently reducing the level of ROS that forms after CTN treatment (Figure 3).

Resveratrol can stimulate or inhibit apoptotic signaling [56–58,60–63]. The current results show that resveratrol blocks CTN-induced ROS generation and various apoptotic biochemical changes in Hep G2 cells at doses greater than 10 μM (Figures 3, 4 and 5). These findings collectively imply that the cell type specificity and action of resveratrol depend on the treatment protocol (i.e., treatment period and dosage). Clearly, the molecular mechanism of action of resveratrol on cell apoptosis requires further investigation.

JNK participates in numerous cell responses, including entry into apoptosis. Using a JNK-specific inhibitor (SP600125), we demonstrated that CTN-induced caspase-3 activation and apoptosis in Hep G2 cells are mediated by JNK activity (Figure 6). Together with the finding that ROS generation and JNK activation triggered by CTN are blocked by resveratrol, these results support the hypothesis that this compound inhibits CTN-induced apoptotic biochemical changes by suppressing ROS formation and JNK activation.

In terms of a possible mechanism underlying the involvement of PAK2 in CTN-induced apoptosis, two previous studies showed that transfection of constitutively activated forms of Rac and Cdc42 into cultured cells led to potent activation of JNK [64,65]. Thus, it appears that PAK participates in the Rac/Cdc42-mediated JNK activation pathway as an upstream regulator of JNK. This theory is strongly supported by a study showing that transfection of N-terminal truncated PAK2 and constitutively active PAK1 into cultured cells leads to JNK activation [22,66]. Moreover, recent experiments by our group showed that activated PAK2 is necessary for apoptosis mediated via JNK- and caspase-dependent signaling in citrinin-treated human osteoblasts[29]. In contrast, we show here that JNK functions upstream of PAK2 during CTN-induced apoptosis (Figure 6). The data suggest that these molecules may act either upstream or downstream of each other in the cell apoptotic signaling pathway. Further research is essential to determine the relationship(s) between PAK2 and JNK during apoptosis.

## 3. Experimental Section

### 3.1. Chemicals

Dulbecco’s modified Eagle’s medium (DMEM), citrinin, resveratrol and 2′,7′-dichlorofluorescin diacetate (DCF-DA) were from Sigma (St. Louis, MO, USA). Z-DEVD-AFC and SP600125 were from Calbiochem (La Jolla, CA, USA).

### 3.2. Cell Culture and CTN Treatment

Human Hep G2 cells were cultured in DMEM supplemented with 20% heat-inactivated fetal bovine serum, 100 U/mL penicillin and 100 μg/mL streptomycin. Resveratrol was dissolved in DMSO and stored as 50 mM stock at −20 ^o^C. Hep G2 cells were incubated with the indicated concentrations of resveratrol for 1 h or left untreated. Next, cells were treated with or without the indicated concentrations of CTN in a CO_2_ incubator for another 24 h. As a control group for resveratrol, DMSO was treatment in the same manner to Hep G2 cells. Cells were washed twice with ice-cold PBS and cell lysates were prepared as previously described [11,26].

### 3.3. MTT Assay

The MTT (3-[4,5-dimethylthiazol-2-yl]-2,5-diphenyltetrazolium bromide) test is a colorimetric assay that measures the percentage of cell survival. Following treatment of cells with CTN for 24 h, 100 μL of 0.45g/L MTT solution was added to each well in 96 wells culture plate. Cells were incubated at 37 ^o^C for 60 min to allow color development, and 100 μL of 20% SDS in DMF-H_2_O (1:1) solution was added to each well to halt the reaction. The plates were then incubated overnight at 37 ^o^C to dissolve the formazan products. The results were analyzed by spectrophotometry using an ELISA reader at a wavelength of 570 nm.

### 3.4. Assessment of Apoptosis and Necrosis

Oligonucleosomal DNA fragmentation in apoptotic cells was measured using the Cell Death Detection ELISA^plus^ kit according to the manufacturer’s protocol (Roche Molecular Biochemicals, Mannheim, Germany). Cells (1x10^5^) were treated with or without various concentrations of CTN at 37 ^o^C for 24 h. Spectrophotometric data were obtained by an ELISA reader at 405 nm. Necrosis was assayed by determining the percentage of cells with plasma membranes permeable to propidium iodide, and apoptosis was assayed as by staining with propidium iodide and Hoechest 33342. Cells were incubated with propidium iodide (1 μg/ml) and Hoechest 33342 (2 μg/mL) at room temperature for 10 min. The percentage of apoptotic cells were determined with plasma membrane impermeable to propidium iodide and condensed/fragmented nuclei stained with Hoechst 33342 with fluorescent microscope. In each experiment 7–10 independent fields (~600–1,000 nuclei in total) were counted per each condition. As a second index of necrosis, we examined the lactate dehydrogenase (LDH) activity present in the culture medium [67]. Cells were cultured in 96-well microtiter dishes containing 100 μL of medium per well in the presence or absence of the indicated concentrations of CTN for 24 h, and the absorption values at 490 nm were determined with an ELISA reader (Promega, Madison, WI, USA). Control blanks consisted of test substances mixed with medium in the absence of cells.

### 3.5. ROS Assay

ROS were measured in arbitrary units using the 2′,7′-dichlorofluorescein diacetate (DCF-DA) fluorescent dyes. Briefly, cells (1.0×10^6^) were incubated in 50 μL PBS containing 20 μM DCF-DA for 1 h at 37 ^o^C, and relative ROS units determined with a fluorescence ELISA reader (excitation at 485 nm, emission at 530 nm). An aliquot of each cell suspension was lysed, and the protein concentrations were determined using a BCA protein assay kit (Pierce, Rockford, IL, USA). The results are expressed as arbitrary absorbance units/mg protein.

### 3.6. JNK Assays

JNK activity, as assayed by the presence of phosphorylated c-Jun protein, was analyzed with the AP-1/c-Jun ELISA kit according to the manufacturer’s protocol (Active Motif, Carlsbad, CA, USA). AP-1 heterodimeric complexes in cellular nuclear extracts were collected by binding to a consensus 5′-TGA(C/G)TCA-3′ oligonucleotide coated on a 96-well plate. The phospho-c-Jun was assayed using a phospho-c-Jun primary antibody and a secondary horseradish peroxidase-conjugated antibody in a colorimeric reaction.

### 3.7. Detection of Mitochondrial Membrane Potential (MMP)

Hep G2 cells were plated and grown on 96-well plates for 24 h, and then incubated with various concentrations of CTN for 24 h. The cells were then separately exposed to the fluorescent dyes, DiOC6(3) (40 nM/well), for 15 min, and fluorescence was measured with a plate spectrofluorometer (excitation: 485 nm; Emission: 535 nm).

### 3.8. Caspase Activity Assays

Caspase-9 activities were assayed using the Colorimetric Caspase-9 Assay Kit (Calbiochem, CA). Caspase-3 activity was measured using the Z-DEVD-AFC fluorogenic substrate, as previously described [30].

### 3.9. Immunoprecipitation and PAK2 Activity Assay

Before immunoprecipitation, cell extracts were diluted to equal protein concentrations (1.0 mg/mL) with cell lysis solution. For immunoprecipitation of the C-terminal catalytic fragment of PAK2, 0.5 mL of cell extract was incubated with 10 μL of anti-PAK2 antibody (200 μg/mL) at 4 °C for 1.5 h, and then further incubated with 40 μL of Protein A-Sepharose CL-4B (30%, v/v) for 1.5 h with shaking. The immunoprecipitates were collected by centrifugation, washed three times with 1 mL of Solution A (20 mM Tris/HCl, pH 7.0, and 0.5 mM DTT) containing 0.5 M NaCl, and resuspended in 40 μL of Solution A. For measurement of PAK2 activity, the immunoprecipitates were incubated in a 50 μL mixture containing 20 mM Tris/HCl, pH 7.0, 0.5 mM DTT, 0.2 mM [γ-p^32^]ATP, 20 mM MgCl_2_ and 0.1 mg/ml myelin basic protein at room temperature for 10 min with shaking. For determination of ^32^P incorporation into the MBP protein, 20 μL of each reaction mixture was spotted onto Whatman P81 paper (1 × 2 cm), which was then washed with 75 mM phosphoric acid and processing as previously described [68].

### 3.10. Inhibition of PAK2 by Anti-Sense Oligonucleotides

The experimental design of PAK2 antisense experiment is followed our previous study [25]. Our previous experiments demonstrated that cells were incubated with the anti-sense or sense oligonucleotide against PAK2 for 3 days and can be seen that the PAK2 decreased markedly (~50%) in the anti-sense oligonucleotide but not the sense oligonucleotide-treated cells as compared to the control cells. In brief, PAK2 sense (5′-ATC ATG TCT GAT AAC GGA GAA) and anti-sense (5′-TTC TCC GTT ATC AGA CAT GAT) oligonucleotides, representing amino acids −1 to +7 of human PAK2, were obtained from Life Technologies (Grand Island, NY, USA). The oligonucleotides were synthesized under phosphorothioate-modified conditions, purified by HPLC, and dissolved in 30 mM HEPES buffer, pH 7.0. For transfections, cells grown in 60 mm culture dishes were incubated at 37 °C in 1 mL of Opti-MEM I medium (modified Eagle’s minimum essential medium buffered with HEPES and sodium bicarbonate), containing lipofectAMINE4 (12 μg) and oligonucleotides (70 μM) for 72 h (all reagents from Life Technologies). The cells were then subjected to CTN treatment, and the cell extracts were analyzed as described above.

### 3.11. Statistics

Data were analyzed using one-way ANOVA and t-tests, and are presented as means ± SD. Data were considered statistically significant at *P*<0.05.

## 4. Conclusions

This study demonstrates that CTN induces various apoptotic biochemical changes through ROS generation and the mitochondria-dependent apoptotic signal pathway in Hep G2 cells. Resveratrol effectively blocks CTN-induced biochemical changes, including ROS generation, JNK activation, loss of MMP, activation of caspase-3 and PAK2, and DNA fragmentation. Our findings support the theory that resveratrol aids in decreasing the toxic effects of CTN via its antioxidant properties.
